# ANCA vasculitis expands the spectrum of autoimmune manifestations of activated PI3 kinase δ syndrome

**DOI:** 10.3389/fped.2023.1179788

**Published:** 2023-05-19

**Authors:** Amika K. Sood, Olivia Francis, Stephen A. Schworer, Steven M. Johnson, Benjamin D. Smith, Paul B. Googe, Eveline Y. Wu

**Affiliations:** ^1^ Division of Rheumatology, Allergy, and Immunology, Department of Internal Medicine, The University of North Carolina, Chapel Hill, NC, United States; ^2^ Division of Allergy/Immunology, Department of Pediatrics, The University of North Carolina, Chapel Hill, NC, United States; ^3^ Department of Pathology and Laboratory Medicine, The University of North Carolina, Chapel Hill, NC, United States; ^4^ Division of Pediatric Radiology, Department of Radiology, The University of North Carolina, Chapel Hill, NC, United States; ^5^ Dermatopathology, Department of Dermatology, The University of North Carolina, Chapel Hill, NC, United States; ^6^ Division of Rheumatology, Department of Pediatrics, The University of North Carolina, Chapel Hill, NC, United States

**Keywords:** activated PI3 kinase delta syndrome, autoimmune, ANCA vasculitis, immunodeficiency, inborn errors of immunity, immune dysregulation, leniolisib

## Abstract

Activated phosphoinositide 3-kinase δ syndrome (APDS) is a combined immunodeficiency with a broad clinical phenotype, including not only an increased propensity for sinopulmonary and herpesviruses infections but also immune dysregulation, such as benign lymphoproliferation, autoimmunity, and malignancy. Autoimmune complications are increasingly recognized as initial presenting features of immune dysregulation in inborn errors of immunity (IEIs), including APDS, so awareness of the spectrum of autoimmune features inherit within these disorders is critical. We present here a patient vignette to highlight cutaneous antineutrophil cytoplasmic antibody (ANCA) vasculitis as an underrecognized autoimmune manifestation of APDS. The genetic defects underlying APDS result in increased PI3Kδ signaling with aberrant downstream signaling pathways and loss of B- and/or T-cell immunologic tolerance mechanisms, which promote the development of autoimmunity. An understanding of the molecular pathways and mechanisms that lead to immune dysregulation in APDS has allowed for significant advancements in the development of precision-medicine therapeutics, such as leniolisib, to reduce the morbidity and mortality for these patients. Overall, this case and review highlight the need to maintain a high index of suspicion for IEIs, such as APDS, in those presenting with autoimmunity in combination with a dysregulated immune phenotype for prompt diagnosis and targeted intervention.

## 1. Introduction

Inborn errors of immunity (IEIs) manifest with broad clinical phenotypes that include not only immunodeficiency with increased propensity for infections but also immune dysregulation, such as benign lymphoproliferation, autoimmunity, and malignancy. Non-infectious manifestations were the initial presenting feature in 25% of IEI patients in the European Society for Immunodeficiencies Registry, which includes over 16,000 entries (1). Autoimmune and autoinflammatory complications, in particular, are increasingly recognized as initial presenting features of immune dysregulation in monogenic immunodeficiency disorders (1, 2). This highlights the need for awareness of the spectrum of autoimmune features inherent within IEIs for early diagnosis and management of these patients.

This is no exception for the combined immunodeficiency activated phosphoinositide 3-kinase δ syndrome (APDS) first described in 2013 (3). In the last decade, over 250 APDS patients have been identified, leading to a greater appreciation of its varied infectious, lymphoproliferative, autoimmune, autoinflammatory, malignant, atopic, and neuropsychiatric manifestations (4, 5). Autoimmunity may affect one-fourth to one-third of APDS patients, with cytopenias reported most frequently (4, 6, 7) and even as a presenting manifestation (2, 8). While vasculitis is a known clinical manifestation of some IEIs (9), it remains an underrecognized feature of APDS. Here, we utilize a patient vignette to highlight cutaneous antineutrophil cytoplasmic antibody (ANCA) vasculitis among the spectrum of autoimmune manifestations of APDS. We then review the pathophysiology, immunological parameters, clinical manifestations, and treatment of APDS, with a particular focus on autoimmunity.

## 2. Case presentation

**Figure 1:**
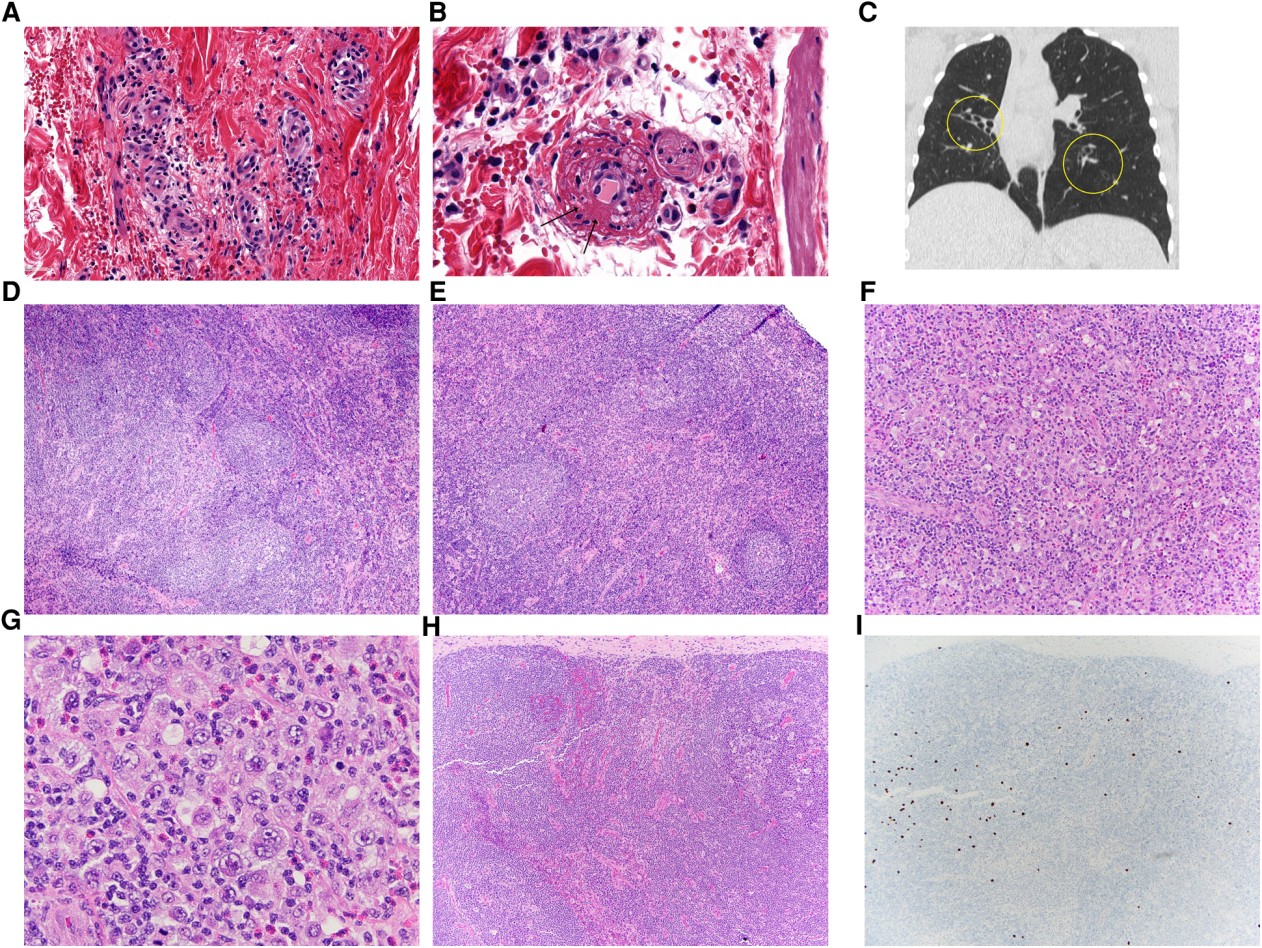
(**A**,**B**) Her skin biopsy has features suggestive of leukocytoclastic vasculitis with dermal capillary-sized blood vessels that are surrounded by lymphocytes, neutrophils, eosinophils and extravasated red blood cells. A capillary (**B**) has fibrin within its wall (arrows), a diagnostic finding for vasculitis. There are adjacent lymphocytes, neutrophils, eosinophils and extravasated red blood cells as expected with a leukocytoclastic vasculitis. (**C**) Coronal HRCT image in lung window demonstrating tubular bronchiectasis and lobar atelectasis of the right middle lobe and also left lower lobe bronchiectasis. (**D**,**E**) The initial cervical lymph node biopsy showed unusual reactive changes, including atypical follicular hyperplasia with poorly-formed and disrupted germinal centers, attenuation of the follicular mantle zones, and irregular expansion of the marginal zone. (**F**,**G**) Her subsequent axillary node showed architectural effacement and characteristic findings of classic Hodgkin lymphoma, with a mixed inflammatory background with small lymphocytes, histiocytes, and eosinophils, and scattered large Hodgkin/Reed-Sternberg cells (**G**). (**H**,**I**) Her follow-up inguinal node biopsy showed intact architecture with reactive features, again with poorly-formed germinal centers (**H**). Scattered EBV-positive cells (**I**) were also seen upon examination by EBV *in situ* hybridization.

A 16-year-old African American female was hospitalized with new-onset petechial rash and palpable purpura. A skin biopsy demonstrated superficial dermal perivascular lymphocytes, neutrophils, eosinophils, and extravasation of red blood cells. Staining of vessels in the dermal papillary tips was positive for C3 and fibrinogen, suggestive of vasculitis (Figures 1A,B). Laboratory evaluation revealed a positive ANCA with cytoplasmic staining (c-ANCA) and elevated proteinase 3 ANCA (PR3-ANCA) along with a positive rheumatoid factor and antinuclear antibody (ANA, titer 1:160) with speckled and cytoplasmic patterns. Testing was negative for anti-double stranded DNA (anti-dsDNA) and extractable nuclear antigen antibodies. C3 and C4 complement levels ranged from just below to just above the lower limit of normal (Table 1). Coagulation studies were not indicative of an underlying thrombotic disorder. As part of an evaluation for other organ involvement, a high-resolution computed tomography scan (HRCT) of the chest was obtained and demonstrated central predominant bronchiectasis and scattered ground glass opacities (Figure 1C). The patient did not have other systemic vasculitis manifestations, and she was diagnosed with cutaneous ANCA vasculitis that resolved without intervention after 6 months despite persistently elevated PR3-ANCA.

**Table 1:** Immunological workup for patient case: abnormal values are in bold.

Lab (reference range)	Lab value
ANA (negative)	**1:160 speckled, cytoplasmic pattern**
Double stranded DNA antibody (negative)	Negative
ENA antibody (<0.7)	0.4
Rheumatoid factor (0–15)	**44.3 IU/ml**
ANCA IFA (negative)	**Positive, cytoplasmic pattern**
MPO quantitative (<21)	8.8 U/ml
PR3 quantitative (<21)	**78.6 U/ml**
C3 (88–171)	89 mg/dl
C4 (15–48)	**14.6 mg/dl**
IgG (600–1,700)	**4,123 mg/dl**
IgM (35–290)	**336 mg/dl**
IgA (40–400)	262.3 mg/dl
IgE (2–537)	**1,631 IU/ml**
Absolute CD3 T cells (915–3,400)	732 cells/µL
% of lymphocytes (61–86)	62%
Absolute CD4 T cells (510–2,320)	**342 cells/µl**
% of lymphocytes (34–58)	**29%**
Absolute CD8 T cells (180–1,520)	378 cells/µl
% of lymphocytes (12–38)	32%
CD8/CD45RA naive T cells as % of total T cells (61–91)	**37%**
CD4/CD45RA naive T cells as % of total T cells (33–66)	**7%**
CD8/CD45RO memory T cells as % of total T cells (4–23)	**63%**
CD4/CD45RO memory T cells as % of total T cells (18–38)	**93%**
Absolute CD19 B cells (105–920)	142 cells/µl
% of lymphocytes (7–23)	12%
Absolute CD16/56 NK cells (15–1,080)	283 cells/µl
% of lymphocytes (1–27)	24%
Transitional B cells (CD19 + CD38 + IgM+) as % of total B cells (7.6–38.6)	**63.1%**
Mature B cells (CD19 + CD21+) as % of total B cells (94.5–99.8)	**82.5%**
Immature B cells (CD19+ CD21−) as % total B cells (0.2–5.5)	**18.1%**
Class Switched B cells (CD19 + CD27 + IgD − IgM−) as % total B cells (1.9–30.4)	9.9%

Notable prior history included developmental delay, seizures, recurrent infections, and lymphadenopathy. Between the ages of 2–10 years, she experienced recurrent sinusitis and otitis media requiring tonsillectomy, adenoidectomy with two revisions, and tympanostomy tube placement with yearly revisions between ages 7–10 years. Otitis media episodes continued despite these surgical interventions. Her infectious history was also notable for hospitalization at 2-years-old for cervical lymphadenitis attributed to Epstein-Barr virus (EBV) and at 3-years-old for a vesicular rash due to varicella zoster virus (VZV) infection.

Chronic, unexplained lymphadenopathy first developed at age 4 years. She underwent an excisional biopsy of a deep right cervical lymph node, which demonstrated follicular hyperplasia with poorly formed germinal centers, mantle zone attenuation, and an irregularly expanded marginal zone (Figures 1D,E). Routine flow cytometric analysis utilizing cell-surface specific antibodies did not identify overt evidence of aberrant B-cell or T-cell populations; the CD4:CD8 ratio was noted to be 1.8:1. Around 14-years-old, she also developed recurrent axillary lymphadenopathy and/or infectious adenitis with response to antimicrobials.

Given the discovery of bronchiectasis on HRCT chest and previous history of lymphadenopathy and recurrent sinopulmonary and herpesvirus infections as noted above, a thorough immune evaluation (Table 1) was conducted. This demonstrated elevated IgM and IgG levels, normal IgA levels, and low CD3+ T-cells, CD4+ T-cells, and CD4 + CD45RA+ and CD8 + CD45RA+ naïve T-cells. Absolute values of CD8+ T-cells, CD19+ B-cells, CD16/56+ NK-cells were normal. B-cell phenotyping showed an increased percentage of transitional B-cells (CD19 + CD38 + IgM+) and decreased percentage of CD21+ mature B-cells with normal percentages of class-switched memory B-cells (CD19 + CD27 + IgD−IgM−). Appropriate antibody titers were demonstrated to T-dependent (tetanus and diphtheria) and T-independent antigens (pneumococcus). Finally, lymphocyte proliferative responses to phytohemagglutinin and pokeweed mitogens were normal.

Given her clinical history and immunophenotype, there was a high degree of suspicion for a monogenic IEI. Genetic sequencing revealed a heterozygous mutation in the *PIK3CD* gene at c.3061G > A encoding the known pathogenic E1021K variant and confirming a diagnosis of APDS type I (3). Immunoglobulin replacement therapy (IRT) at 300 mg/kg/month was initiated with marked improvement in frequency and severity of infections. At 17-years-old, she developed right axillary lymphadenitis refractory to antimicrobials. She underwent a right axillary lymph node biopsy with histopathology consistent with classic Hodgkin lymphoma (CHL, Figures 1F,G). Cytogenetic analysis observed no clonal chromosomal abnormality while flow cytometry did not reveal a monotypic B-cell population or an aberrant T-cell population. Marked improvement in lymphadenopathy occurred following treatment with a standard chemotherapy regimen. However, over the next year, she developed progressive non-malignant lymphadenopathy (Figures H,I) with adenitis. Thus, sirolimus was initiated with notable improvement in adenopathy.

## 3. APDS: genetics, pathophysiology, and immunocellular phenotype

Phosphoinositide-3 kinases (PI3K) are a group of lipid kinases critical to the normal functioning of widespread and diverse cellular processes. Of the three class IA PI3 kinases, PI3Kδ, a heterodimer consisting of a catalytic and regulatory subunit, is preferentially expressed in immune cells and functions downstream of varied cell-surface receptors, including cytokine receptors, T- and B-cell antigen receptors, costimulatory molecule receptors, growth receptors, and toll-like receptors. Signaling pathways downstream of PI3Kδ activation then orchestrate the growth, activation, proliferation, differentiation, and survival of immune cells (10–12).

APDS results from disruption of this tightly controlled signaling cascade through autosomal dominant gain-of-function (GOF) mutations in either the *PIK3CD* gene encoding the p110δ catalytic subunit (APDS type I, APDS1) or the *PIK3R1* gene encoding the p85α, p55α, and p50α regulatory subunits (APDS type 2, APDS2) (3, 13–15). The most common heterozygous mutation found in the *PIK3CD* gene, accounting for 85% of APDS1 cases, results in a missense mutation at c.3061G > A (p. E1021K) and is not found in the healthy population (3, 4). A heterozygous exon-skipping mutation in the *PIK3R1* gene, resulting in deletion of amino acids 434–475 in the p85α regulatory subunit, accounts for 79% of APDS2 cases (4, 14, 15).

These mutations result in increased PI3Kδ pathway activity with upregulated phosphorylation of phosphatidylinositol-4,5-bisphosphate to form phosphatidylinositol-3,4,5-trisphosphate (PIP3). PIP3 in turn enhances activation of the AKT serine-threonine kinases (3) with a number of downstream effects on cellular immune processes. First, phosphorylated AKT phosphorylates a variety of transcriptional regulators, including FOXO1, NF-κB, and BACH2. Inactivation of FOXO1, specifically, results in decreased expression of the proapoptotic BCL-2 family of proteins as well as proteins involved in immunoglobulin gene recombination, such as RAG1 and RAG2 (11, 12). Activated AKT also impacts cell survival and migration by inhibiting glycogen synthase kinase 3β, resulting in upregulation of the anti-apoptotic BCL-2 family member MCL1 and inactivation of NFAT (12). Finally, the serine/threonine kinase mTOR activity is upregulated through phosphorylation and inactivation of the inhibitory proteins, tuberous sclerosis complex (TSC) 1/2 by activated AKT. In turn, mTOR enhances activation of ribosomal S6 kinase, significantly impacting regulation of protein synthesis and cell metabolism (12). In contrast to many immunodeficiencies, the net result in APDS is hyper-activation of signaling rather than absence of signaling (11).

These dysregulated signaling pathways in APDS have diverse effects on T- and B-cells. B-cells demonstrate increased apoptosis along with impaired maturation in the bone marrow beyond the transitional B-cell stage (16–18). Intact B-cell populations demonstrate impaired class-switch recombination (CSR) and somatic hypermutation (SMH) associated with increased plasmablast differentiation to IgM-secreting B-cells (17, 18). Progressive B-cell lymphopenia is seen in up to 75% of APDS patients with skewing towards greater percentages of transitional B-cells, as demonstrated in our patient, but reduced class-switched memory B-cells (4, 7, 15). More than half of patients also demonstrate elevated IgM levels but low levels of IgG and IgA (4, 7, 15) along with poor antibody responses to vaccinations (7) secondary to defective CSR.

Like B-cells, T-cells also demonstrate increased apoptosis (19, 20). While increased effector CD8+ T-cell populations are seen, these cells are marked by increased expression of activation markers CD160, CD244, and PD-1, indicative of premature immunosenescence and exhaustion with reduced cytotoxicity (19–21). Differentiation into central memory T-cells is also impaired secondary to lack of expression of proteins critical to this process (20). Expansion of follicular helper T-cells (TfH) occurs at the expense of peripheral CD4+ T-cells (22). To this effect, APDS patients can have an inverted CD4:CD8 ratio, and CD4+ T-cell lymphopenia affects up to two-thirds of patients. While CD8+ T-cell lymphopenia occurs less frequently, effector CD8+ T-cells are typically increased and associated with reduction in naïve T-cells (4, 7, 15).

## 4. APDS: non-autoimmune clinical manifestations

Disease progression in APDS classically involves chronological development of infectious, benign lymphoproliferative, autoimmune, and malignant complications (4, 6) with median age of onset of occurrence of <1 year, 3 years, 10.5 years, and 18 years, respectively (4). Recurrent upper and lower respiratory infections affect almost all patients (4–6) with rates of pneumonia in up to 70% (5) and concomitant bronchiectasis in 28%–51% (4–6). Lymphadenitis, cutaneous cellulitis/abscesses, chronic mucocutaneous candidiasis, and localized granulomatous disease following bacille Calmette-Guerin vaccination are the more commonly reported non-respiratory infections. More than one-third of patients may develop acute herpesvirus infections with EBV, cytomegalovirus, herpes simplex virus 1/2, human herpes virus 6, and VZV with development of chronic EBV in a quarter of patients (4–6).

Benign lymphoproliferation occurs in up to 87% of patients, manifesting as lymphadenopathy, splenomegaly, hepatomegaly, tonsillar/adenoid hypertrophy, and nodular hyperplasia within gastrointestinal and respiratory mucosa. Diffuse large B-cell lymphoma, CHL, and marginal zone B-cell lymphoma are the most common forms of malignant lymphoproliferation with an increased risk of development in those with previous or chronic EBV infection (4–6). Patients with APDS2 also have an increased propensity for growth and developmental delay as well as neuropsychiatric disorders compared to those with APDS1 (4, 6). Conversely, atopy is seen in higher rates in APDS1 vs. APDS2 patients (5).

## 5. APDS: autoimmune clinical manifestations

In the largest systematic review of APDS patients to date, autoimmune features were reported in 28% of 243 total patients (4). Table 2 summarizes the spectrum of autoimmunity described amongst APDS patients reported in the literature. Autoimmune cytopenias and gastrointestinal manifestations are significantly more common than renal or rheumatological diseases or endocrinopathies (4). Variable autoantibody production in APDS patients has also been reported, including ANA, perinuclear ANCA (p-ANCA), PR3-ANCA, and anti-Smith, anti-Ro/SSA, anti-cardiolipin, anti-dsDNA, anti-ribonucleoprotein, and anti-smooth muscle antibodies (16, 23–25, 37, 38, 40, 42). Correlation between these autoantibodies and reported autoimmunity was often not clearly delineated.

**Table 2:** Spectrum of autoimmune manifestations in APDS.

Autoimmune Manifestation	Reference
Cytopenia (immune thrombocytopenic purpura, autoimmune hemolytic anemia, neutropenia, Evans Syndrome, pancytopenia)	(3, 5–8, 14–16, 23–32)
Inflammatory bowel disease	(5, 14, 25, 27, 30–33)
Sclerosing cholangitis	(7, 25, 33, 34)
Hepatitis	(6, 15)
Pancreatic Insufficiency	(7, 27)
Inflammatory Arthritis	(6, 7, 14, 15, 32, 35, 36)
Thyroiditis	(5–7, 27, 30, 37)
Nephritis	(6, 7, 23–25, 36, 38)
Pericarditis	(7, 31, 39)
Parotiditis	(30)
Systemic Lupus Erythematous	(23, 32)
Sjogren's Syndrome	(24)
Vasculitis	
• Digital Vasculitis	(40)
• Cutaneous Vasculitis	(41)
• Granulomatous polyangiitis	(42)
• Vertebral vasculitis	(5)
Autoantibodies	(16, 23–25, 37, 38, 40, 42)

As noted in Table 2, vasculitis is an infrequent autoimmune feature amongst APDS patients. In 2019, Hong et al. reported chronic vasculitis affecting the fingers and toes of a pediatric APDS2 patient who was interestingly found to have a C1q deficiency secondary to increased consumption rather than intrinsic production defect or anti-C1q antibodies (40). The etiology of vasculitis was postulated to be secondary to low C1q similar to the lupus-like vasculitis seen in genetic C1q deficiency (43). In 2021, Lu et al. described two pediatric patients with recurrent lung infections, sinusitis, hematuria, and positive PR3-ANCA antibodies who initially were diagnosed with granulomatosis with polyangiitis but were later genetically confirmed to have APDS1 (42). Similarly, APDS1 was confirmed in a 7-year-old boy with recurrent sinopulmonary infections, bronchiectasis, lymphoproliferation, and hematuria after an initial diagnosis of ANCA-associated vasculitis three years earlier (38). No cutaneous vasculitic manifestations were reported in these patients (38, 42). There was a single report of vertebral vasculitis in an APDS cohort from the United States Immunodeficiency Network (USIDNET) Registry (5). Most recently, Larrauffie et al. reported leukocytoclastic vasculitis associated with concomitant toxoplasmosis infection with recurrence despite treatment of toxoplasmosis (41). The presence of autoantibodies in this patient was not discussed. Our report of cutaneous ANCA vasculitis adds to this small, but growing, patient cohort, emphasizing that vasculitis may be an underrecognized APDS manifestation.

## 6. APDS: treatment

Efforts to mitigate the infectious, lymphoproliferative (benign and malignant), and autoimmune complications of APDS have centered on combination use of antibiotic prophylaxis, IRT, immunomodulators, selective PI3Kδ inhibitors, and hematopoietic stem cell transplant (HSCT) (44). While two-thirds of patients may be treated with antibiotic prophylaxis, the vast majority still require long-term IRT given the predominance of sinopulmonary infections, inability to generate an adequate specific antibody response, and variable IgG production (6, 7, 15). Since these treatments do not alter the course of non-infectious complications, immunosuppressive therapies are necessary in most cases (5, 6). Various immunomodulators have been utilized, including corticosteroids, rituximab, cyclosporine, azathioprine, and mycophenolate with greatest use of sirolimus to target the over-activation of mTOR observed in APDS (6). Improvements in T-cell populations have been demonstrated with sirolimus (13) along with reduction in benign lymphoproliferation (6, 13, 15). However, side effects and variable efficacy, particularly for autoimmune manifestations (6, 15), has limited its use and highlight the need for improved therapeutic options.

Selective PI3Kδ inhibitors have been the focus for this search for treatments with greater efficacy and more favorable side effect profiles. Leniolisib (CDZ173) is a potent oral small molecule inhibitor of the p110δ catalytic subunit with promising results in APDS patients. Rao et al. recently published results from the global, phase 3, randomized (2:1), placebo-controlled trial of leniolisib in 31 APDS patients (45). Leniolisib treatment significantly increased naïve B-cell percentages and reduced transitional B-cells, plasmablasts, CD8+ senescent CD57+ and PD-1+ T-cells, and IgM levels. Leniolisib treatment also normalized baseline inverted CD4:CD8 T-cell ratios and decreased systemic inflammatory makers and CXCL13, a chemokine marker for TfH cell activity and aberrant lymphocyte trafficking to lymphoid follicles. Both lymphadenopathy and splenomegaly improved with leniolisib in addition to 82% of baseline cytopenias vs. 60% in the placebo group. While not statistically significant compared to placebo, greater improvements in overall well-being was reported by patients receiving leniolisib. Leniolisib was well-tolerated over the 12-week study period. Similar immunologic and clinical improvements were also reported in the initial 12-week, open-label, dose-escalation study of leniolisib in 6 APDS patients. Notably, all patients demonstrated decreased lymphoproliferation with increased platelet counts seen in 5/6 patients with prior history of thrombocytopenia, indicating the potential of leniolisib to alter the course of both lymphoproliferation and autoimmunity (46).

Finally, HCST has been pursued in APDS patients with severe, treatment-refractory disease (24, 25). Significant improvements in infectious complications and elimination of IRT need are reported post-HCST with positive effects on both lymphoproliferation and autoimmunity. While overall survival of APDS post-HSCT appears similar to other primary immunodeficiencies (24, 44), transplant-related complications are common and may necessitate re-transplant (24, 25). Whether the need for HSCT in patients with severe APDS will continue with more widespread use of targeted therapy with leniolisib remains to be seen.

## 7. Discussion

Normal immune function depends on balanced PI3Kδ pathway signaling as evidenced by the immunodeficiency and immune dysregulatory features that develop from heterozygous GOF mutations in PI3Kδ. The clinical heterogeneity of APDS is likely due to a complex interplay between environmental and pathogen exposures and genetic predisposition. While APDS patients have wide-ranging clinical phenotypes from being asymptomatic to substantially severe (3, 7), the overall disease burden and healthcare utilization are fairly significant often with need for multiple surgical interventions and combination of treatment modalities (5, 6). The early deaths reported from both disease- and treatment-related complications (7) further highlights the increased morbidity and mortality of APDS patients. Unfortunately, diagnostic delay in APDS is common (5, 37) with many patients initially misdiagnosed with other humoral immunodeficiencies, such as hyper IgM (HIGM) syndrome or common variable immune deficiency (CVID) (4). This underscores the critical importance of recognizing the full spectrum of APDS clinical manifestations for early diagnosis and institution of disease-modifying therapeutics.

Our case highlights cutaneous ANCA vasculitis as an underreported autoimmune manifestation of APDS while also demonstrating well-established features, such as recurrent sinopulmonary and herpesvirus infections, bronchiectasis, lymphadenopathy, increased transitional B-cells, total and naïve T-cell lymphopenia, and dysgammaglobulinemia. Spontaneous resolution of the vasculitis was observed similar to the clinical course of uncomplicated primary cutaneous vasculitis (47) though ANCA positivity remained, indicating that the presence of autoantibodies alone is insufficient for disease manifestation. However, in APDS patients with complicated or systemic vasculitic features, immunomodulatory regimens, such as corticosteroids, hydroxychloroquine, azathioprine, mycophenolate, and cyclophosphamide, have been utilized with variable results (38, 40–42). In one such case, targeting mTOR hyperactivity with sirolimus allowed for improved management of the associated autoimmune vasculitis (41). While ANCA vasculitis associated with APDS may share clinical and histological features with primary ANCA vasculitis, there are too few cases of ANCA vasculitis reported in APDS to thoroughly render comparisons, but understanding the differing molecular underpinnings will aid in a more targeted approach to therapy.

Vasculitis was reported in approximately 1% of the more than 5,000 IEI patients in the USIDNET registry, with the highest rates in interferonopathies and Wiskott–Aldrich syndrome. However, vasculitis occurs in several other immunodeficiencies, including HIGM, CVID, Hyper IgE-syndrome, complement deficiencies, chronic granulomatous disease, agammaglobulinemia, and autoimmune lymphoproliferative syndrome (9). The spectrum of IEIs in which vasculitis occurs emphasizes the complex and sundry pathophysiological pathways that lead to autoimmunity in immunodeficiencies. Proposed dysregulated mechanisms include lymphopenia from defective formation of T- and B-cell antigen receptors, apoptotic defects or impaired clearance of apoptotic debris, breakdown in central or peripheral tolerance checkpoints, impairment of B-cell function (including defective CSR and SHM), increased interferon signature, and early complement deficiencies (48), several of which are seen in APDS. As previously discussed, hyperactive PI3Kδ signaling results in defects in immune cell apoptosis and skewing of lymphocyte subsets with increased IgM-secreting plasma cells (17, 18, 20) and TfH cells (49). This combination of IgM-secreting plasmablasts with impaired CSR and increased TfH cells lead to disorganized germinal center reactions and a loss of tolerance with increased production of autoantibodies, creating a favorable environment for autoimmunity development (49, 50).

DNA sequencing advancements and identification of monogenic defects underlying IEIs has led to a better understanding of the molecular pathways and mechanisms that result in their clinical manifestations. This, in turn, has allowed for significant advancements in developing precision-medicine therapeutics (51), including leniolisib for APDS. While leniolisib has shown early promise in treating the lymphoproliferation and autoimmune cytopenias in APDS and improving dysregulated immune pathways, unanswered questions remain. First, treatment of patients with broader clinical phenotypes will help determine whether leniolisib will effectively treat non-cytopenic autoimmune manifestations. Second, further studies are necessary to elucidate whether leniolisib's ability to reverse hyperactive PI3Kδ signaling can alter the natural course of APDS and prevent disease complications, such as autoimmunity and malignancy, if administered to patients prior to onset of these manifestations. Third, as leniolisib's use becomes widespread and prolonged, monitoring for safety events will be important as PI3Kδ inhibition can increase genomic instability within B-cells (52). Despite these remaining questions, leniolisib has positively altered the treatment landscape for APDS patients. For patients presenting with autoimmunity such as vasculitis in combination with immune dysregulation, it is important to maintain a high index of suspicion for IEIs, such as APDS, and pursue genetic sequencing for prompt diagnosis and targeted intervention.
